# Transcriptome analysis of genes and carbon partitioning pathways involved in high temperature stress resilience in groundnut (*Arachis hypogaea* L.)

**DOI:** 10.1038/s41598-025-15509-4

**Published:** 2025-08-15

**Authors:** Rachana Bagudam, Anurag Mathew, Eswari Kancherla, Sai Rekha Kadirimangalam, Sudheer Kumar S, Prasad Bajaj, Narender Reddy S, Janila Pasupuleti

**Affiliations:** 1 International Crops Research Institute for the Semi-Arid Tropics (ICRISAT), Hyderabad, India; 2 Professor Jayashankar Telangana State Agricultural University (PJTSAU), Rajendranagar, Hyderabad India; 3 Acharya N. G. Ranga Agricultural University (ANGRAU), Guntur, Andhra Pradesh India; 4 Dr. Y.S.R. Horticultural University, West Godavari, India

**Keywords:** Antioxidant, Carbon partitioning, Groundnut, Gene expression, High-temperature stress, Sugars, Biotechnology, Physiology, Plant sciences

## Abstract

For the climate change scenarios where the temperatures during crop growing season are on the rise, tolerance to high-temperature (HT) stress in groundnut, especially during flowering and pod stages, is critical to sustain production in arid and semi-arid regions. To elucidate the regulatory mechanisms underlying the response of groundnuts to HT stress, a heat-tolerant (ICGV 16553) and a heat-sensitive (ICGV 16516) genotypes were identified based on responses in multi-season HT stress field experiments. The tolerant genotype had significantly higher total antioxidant activity and phenol content than the sensitive genotype in response to HT stress at flowering stage. Translocation studies revealed efficient sucrose loading into phloem, and increased glucose levels in the pods are indicative of an active sucrose metabolism in the tolerant genotype at the pod stage under HT stress. Transcriptomic analysis revealed a total of 1631 and 836 differentially expressed genes (DEGs) at flowering and pod stages, respectively. The identified DEGs encoded heat shock proteins, sucrose metabolism enzymes involved in photosynthate synthesis and translocation to sink tissues, transcriptional genes and antioxidants which played a major role in mitigating the effects of HT stress. The genes response in both sensitive and tolerant genotypes at each stage can provide valuable insights for future investigations and the genetic improvement of groundnut for heat tolerance mechanisms.

**Supplementary Information:**

The online version contains supplementary material available at 10.1038/s41598-025-15509-4.

## Introduction

 Groundnut (*Arachis hypogaea* L.) or peanut is cultivated in 113 countries occupying an area of about 30.53 million hectares with a production of 54.23 million metric tons^1^. It is extensively grown in the arid and semi-arid agro-ecologies, where the growth and production is challenged by various biotic and abiotic factors. Climate change is the main threat to yield and quality of groundnut cultivation in these regions due to increased incidences of drought and rising high temperatures^2^. Predictions revealed that the temperature will rise by 1.5°C in the first half of the 2030’s and will make it very difficult to control temperature increases by 2.0°C towards the end of the 21st century^3^. A modeled estimate under the changing climate scenario indicated a change of −34% to + 43% in groundnut production by 2050 across India’s growing regions^4^.

High-Temperature (HT) stress, a consequence of climate change, negatively impacts plant growth, development, and crop yield. The severity, duration, and rate of temperature change have a complex relationship with the impact of HT stress^5^. HT stress affects the plant physiology, biochemistry, and gene regulation pathways^6,7^. Groundnut, with an optimum temperature range of 25–30°C for growth and development, faces adverse effects with the rise in temperature. Reproductive stages like flowering and pod development are sensitive to high temperatures^8,9^. Due to their aerial flowering and underground fruiting habit, groundnuts are vulnerable to both high air and soil temperatures^8^. Soil temperatures above 33°C affect the total dry matter production, partitioning of dry matter into pods, and yield of groundnut^10^. While, air temperatures above 35°C reduce pod yields substantially by 1.5–43.2%^11^ and 15–25%^12^. Yield losses due to high temperatures could be attributed to reduced flower number, poor pollen viability, and smaller seed size because of impaired partitioning of dry matter into pods^10^. The developmental and functional disruptions during the reproductive stage, such as the accumulation of sucrose and starch in pollen grains and damage to male components, are caused by the sensitivity to HT^13^.

HT stress tolerance in groundnut involves a complex interplay of physiological, biochemical, cellular, and molecular mechanisms. Physiological traits include chlorophyll content^14^ and cell membrane stability, which determine the membrane integrity when exposed to high temperatures^14,15^ can be used as surrogate traits to determine HT tolerance^16^. HT stress also affects the relationship between the carbon content in photosynthetic organs, such as leaves (source), and that of the carbon content in seeds (sink), indicating the process of carbon partitioning is HT sensitive^17^. Under HT stress, a study reported that the photosynthates accumulated were not efficiently partitioned into the pods, and genotype differences were observed in groundnut^11^. However, the partitioning of carbon assimilates was documented based on sucrose metabolism, starch accumulation, and the enzyme activity in the leaves, and seeds of common bean^18^ and soybean^19^ under drought stress and in chickpea^20^ under HT stress. Little is known about the sucrose metabolism and starch accumulation in groundnut under HT stress.

At the biochemical level, plants have developed various mechanisms against HT, including the production of phytohormones, antioxidants, and osmo-protectants as an adaptive response^21^. One of the main plant stress responses to HT is the production of reactive oxygen species (ROS), which causes lipid peroxidation, protein oxidation, DNA damage, and destruction of pigments^22,23^. To combat ROS production, HT stress-tolerant plants induce an antioxidant system that acts as a ROS scavenging agent. There are two antioxidant defensive mechanisms in plants, enzymatic (catalase, ascorbate peroxidase, superoxide dismutase etc.) and non-enzymatic (phenolic acids, ascorbic acid etc.) antioxidants^22,24^. Studies showed the role of antioxidant response under abiotic stress conditions like drought stress in groundnut^25^, mungbean^21^ and HT stress in mungbean^13^.

A few attempts were made to reveal the role of heat shock proteins underlying HT stress in groundnut^12,26^. With the sequencing of groundnut tetraploid genome and the advent of recent -omics technologies, it is possible to study the molecular mechanisms underlying HT tolerance^27^. In a recent study, three quantitative trait locus (QTL) clusters (Cluster-1-Ah03, Cluster-2-Ah12, and Cluster-3-Ah20) that harbor more than half of the major QTLs with PVE ranging from 10.1% to 49.5% for various HT stress tolerant traits were identified in groundnut^28^. Fine mapping of such QTL-rich intervals and identification of candidate genes is required for their future use in marker-assisted selection (MAS). One of the most powerful and reliable next-generation sequencing technology, i.e., RNA-Sequencing (RNA-Seq), is widely used to identify the differentially expressed genes (DEGs) under HT stress responses and is reported in several crop species like soybean^29,30^, common bean^31^, lentil^32^, and pigeon pea^33^.

Studies were focused on transcriptome profiling of groundnut under drought conditions^34,35^ and the present study attempts genome-wide transcriptional regulation of groundnut under high-temperature stress. Besides, the study examined antioxidant activity, translocation of photosynthates and gene expression in contrasting genotypes to explore the impact of HT-stress during critical stages, namely flowering and pod-development.

## Results

### Response of tolerant and sensitive genotypes to HT stress

**Fig. 1:**
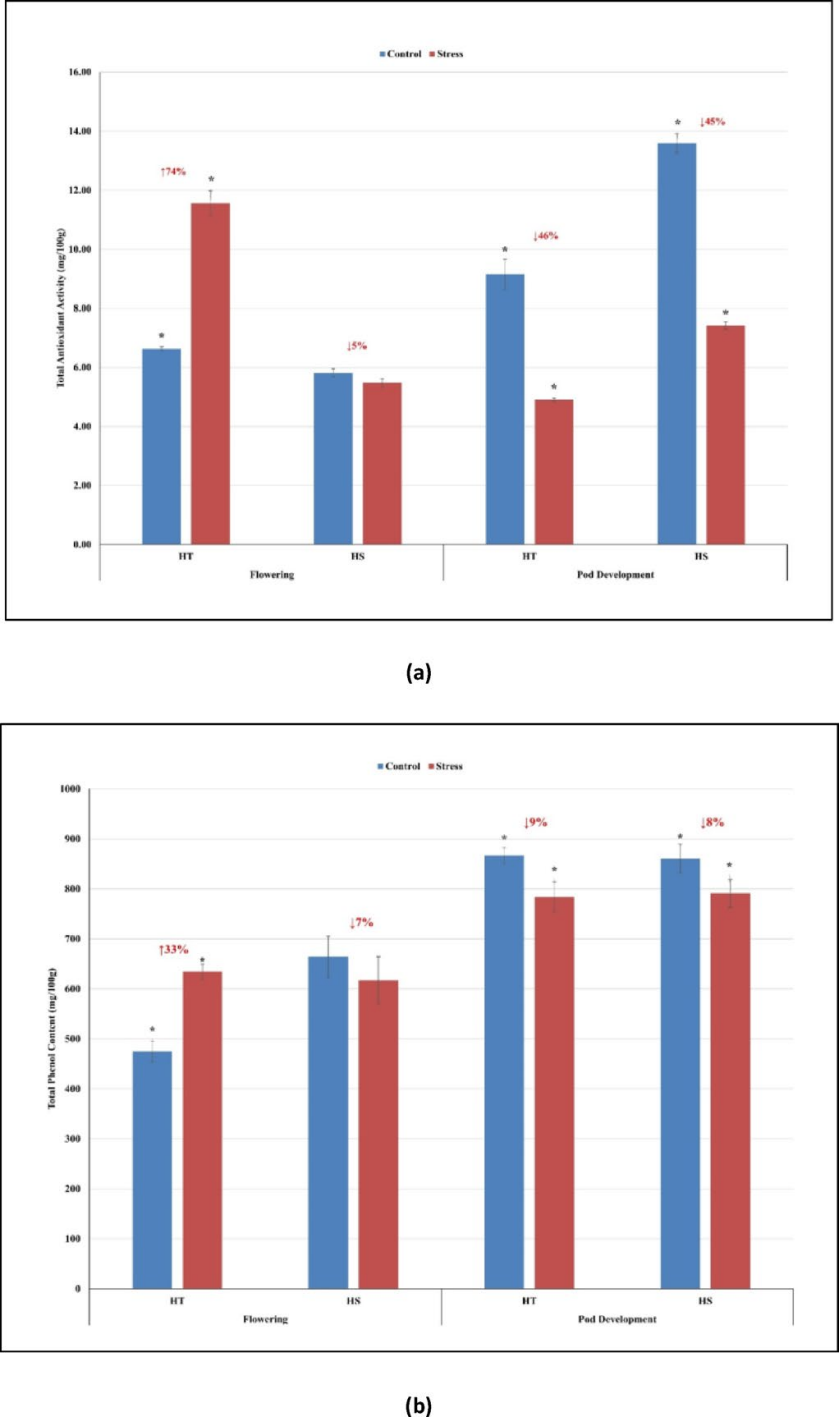
Graphical representation of **(a) **total antioxidant activity and **(b)** total phenol content in heat-tolerant (HT) and heat-sensitive (HS) genotypes under control and stress conditions during flowering and pod development stage. Note: X-axis represents the two genotypes along with stage of sampling, Y-axis represents the total antioxidant activity/total phenol content of HT and HS genotypes. *P<0.05, a significant difference in antioxidant activity/phenol content in HT and HS genotypes under stress compared to control.

Compared to control conditions, Total antioxidant activity (TAA) significantly increased in tolerant genotype (ICGV 16553) by 74% and decreased in sensitive genotype (ICGV 16516) by 5% at the flowering stage (Fig. 1a & Supplementary Table S1). However, TAA declined by 45–46% in both the genotypes at the pod development stage. Total phenol content (TPC) increased in tolerant genotype by 33% and decreased in sensitive genotype by 7% under HT stress compared to control conditions at the flowering stage. However, TPC decreased by 8–9% in both tolerant and sensitive genotypes at the pod development stage (Fig. 1b & Supplementary Table S1).

**Fig. 2:**
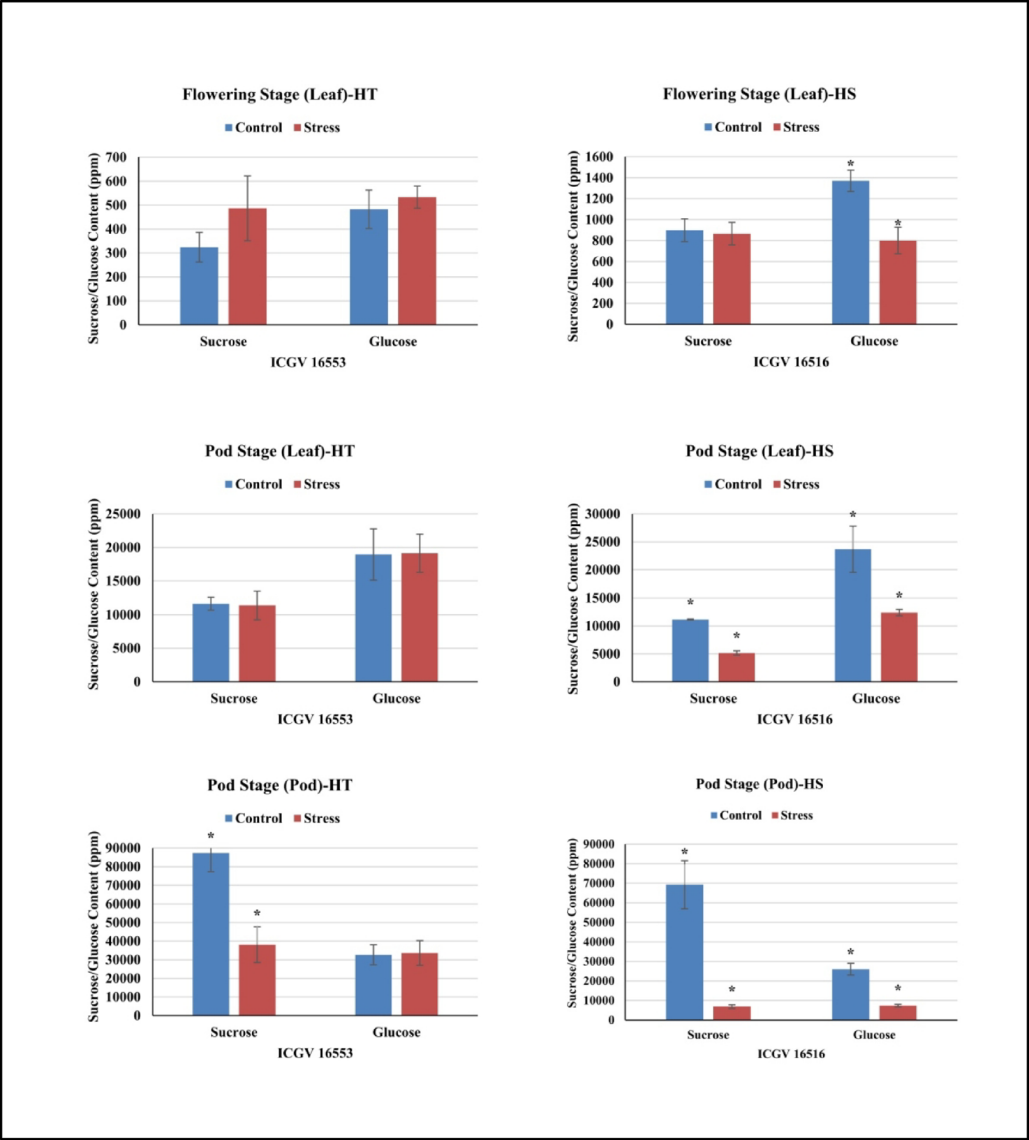
Graphical representation of sucrose (ppm) and glucose (ppm) levels in heat-tolerant (ICGV 16553) and heat-sensitive (ICGV 16516) genotypes under control and stress conditions during flowering and pod development stages. Note: X-axis represents the sugars-sucrose and glucose in ICGV 16553/ICGV 16516 under control and stress conditions, Y-axis represents the quantified amount of sugars in heat-tolerant and heat-sensitive genotypes. *P < 0.05, a significant difference in sugar content in HT and HS genotypes under stress compared to control.

**Fig. 3:**
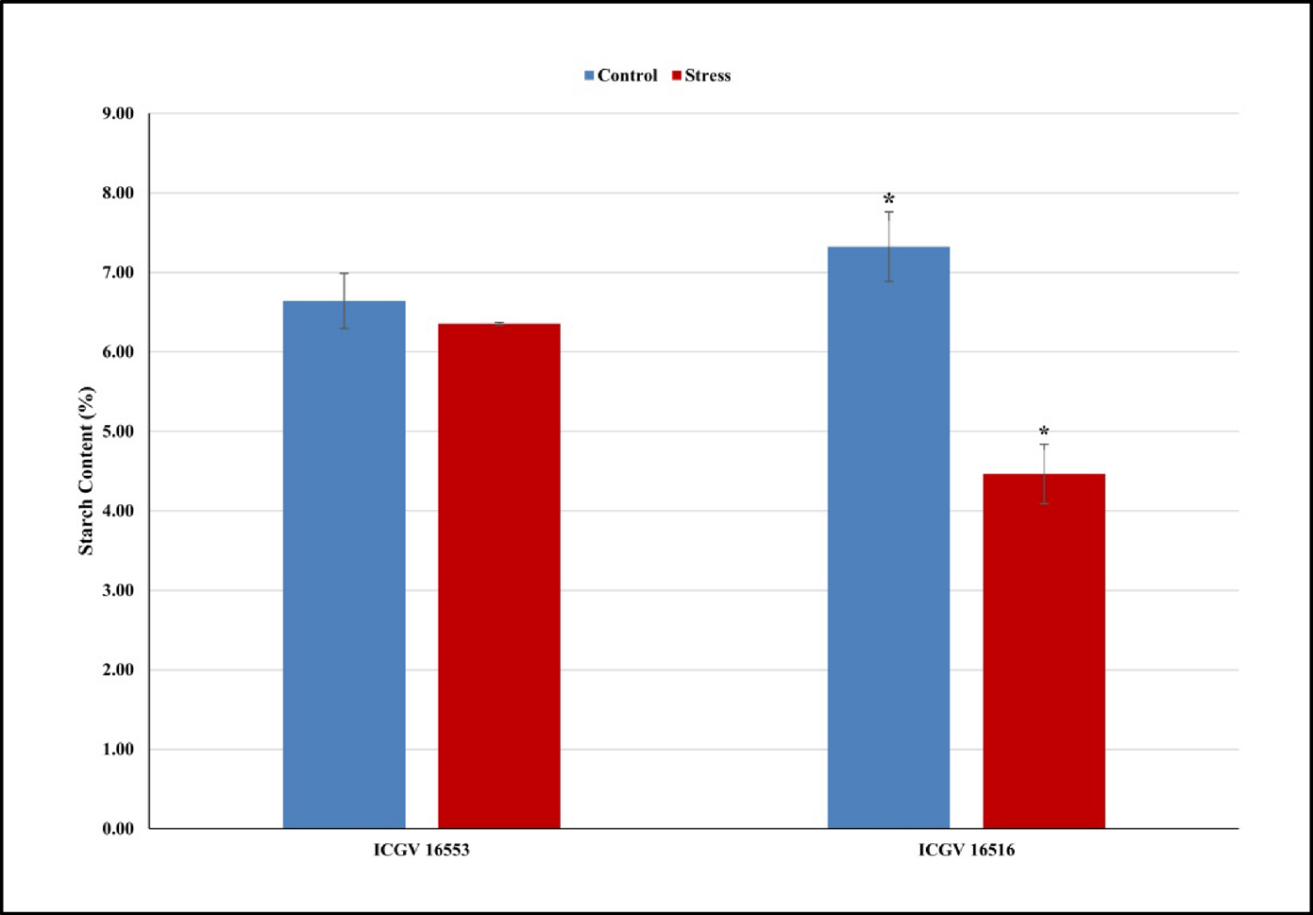
Graphical representation of starch content (%) in the kernels of heat-tolerant (ICGV 16553) and heat-sensitive (ICGV 16516) genotypes under control and stress conditions. Note: X-axis represents the heat-tolerant and heat-sensitive genotypes; Y-axis represents the amount of starch (%). *P < 0.05, a significant difference in amount of starch amount in heat-tolerant and heat-sensitive genotypes under stress compared to control.

Translocation of assimilates from source to sink: In the tolerant genotype (ICGV 16553), both glucose (by 11%) and sucrose (by 50%) content increased in the leaves at the flowering stage under HT stress compared to the control conditions. Compared to the control, the HT stress increased the glucose levels (1% and 3%) and reduced sucrose levels (2% and 56%) in the leaves and pod samples, respectively, at the pod development stage (Fig. 2 & Supplementary Table S2). The accumulation of starch in the harvested kernels of tolerant genotype was 6.6% and 6.3% under control and HT stress conditions, respectively (Fig. 3 & Supplementary Table S3). While, in the sensitive genotype (ICGV 16516), glucose decreased by 42% and sucrose by 4.0% in the leaves at flowering under HT stress compared to the control conditions. Compared to the control, high temperatures greatly reduced the glucose (47% & 71%) and sucrose levels (54% & 90%) at the pod development stage in the leaves and pod samples, respectively (Fig. 2 & Supplementary Table S2). The starch accumulation in sensitive genotype showed a drastic decline of 38% under HT stress compared to control conditions (Fig. 3 & Supplementary Table S3).

### Transcriptome sequencing and assembly

The RNA-sequencing was used to profile the transcriptome of leaf samples including tolerant (ICGV 16553) and sensitive (ICGV 16516) groundnut genotypes at flowering and pod development stages, respectively. 151.9 million reads accounting for 18.7 GB of data were generated. After trimming the low-quality reads, a total of 149.9 million reads, on average 98%, were aligned to the reference genome (cultivar Tifrunner) version gnm2, which is a modified version of the reference genome (cultivar Tifrunner) version gnm1 using the paired-end sequencing (Table 1). In the tolerant genotype, 38.39 and 41.28 million reads were filtered at the flowering and pod development stages, respectively. While, in the sensitive genotype, 38.78 and 31.49 million reads were filtered at the flowering and pod development stages, respectively, under HT stress conditions.

### Differentially expressed genes (DEGs) under HT stress

**Fig. 4:**
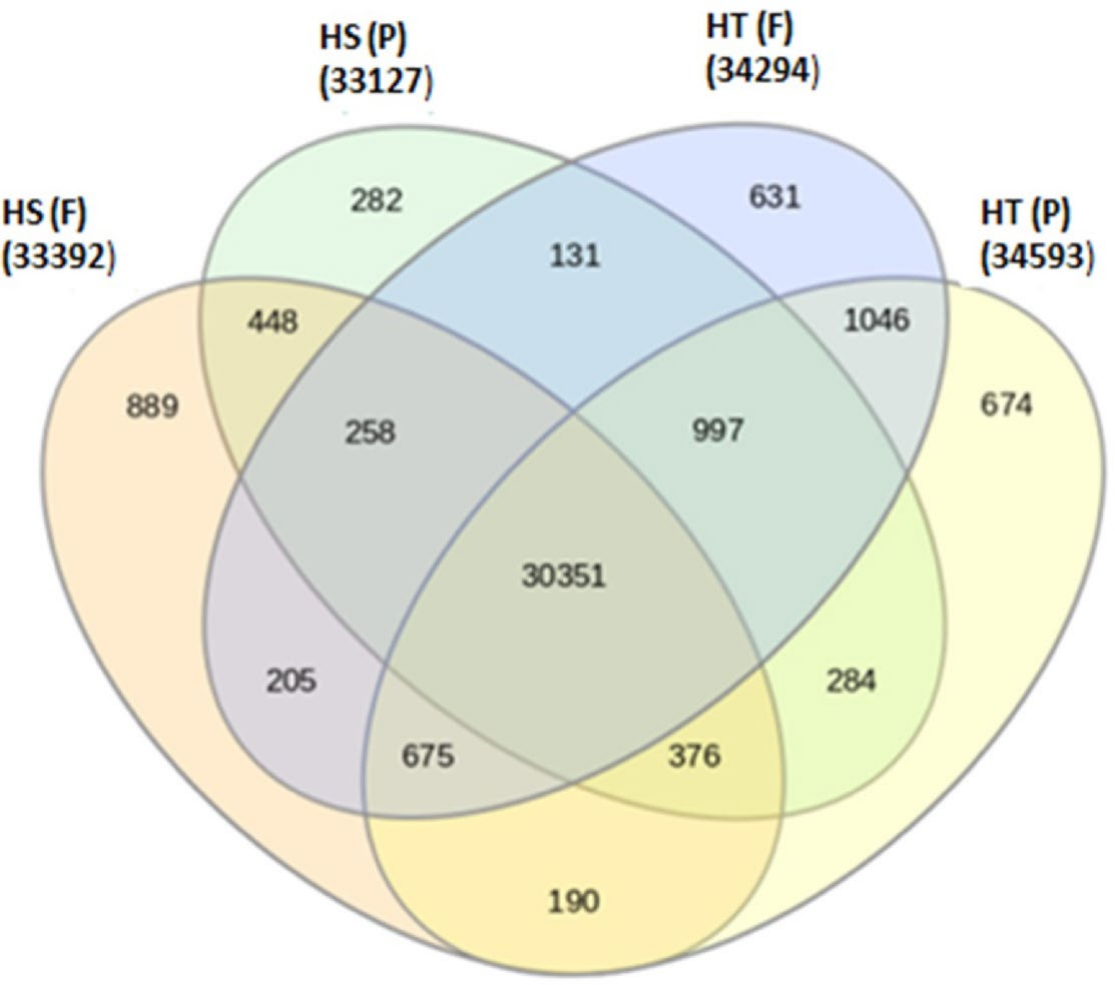
Venn diagram of different genes expressed in tolerant and sensitive genotypes at flowering and pod development stages under high-temperature stress. Note: HS (F)- Heat sensitive line at flowering stage; HS (P)- Heat sensitive at pod development stage; HT (F)- Heat tolerant line at flowering stage; HT (P)- Heat tolerant line at pod development stage.

**Fig. 5:**
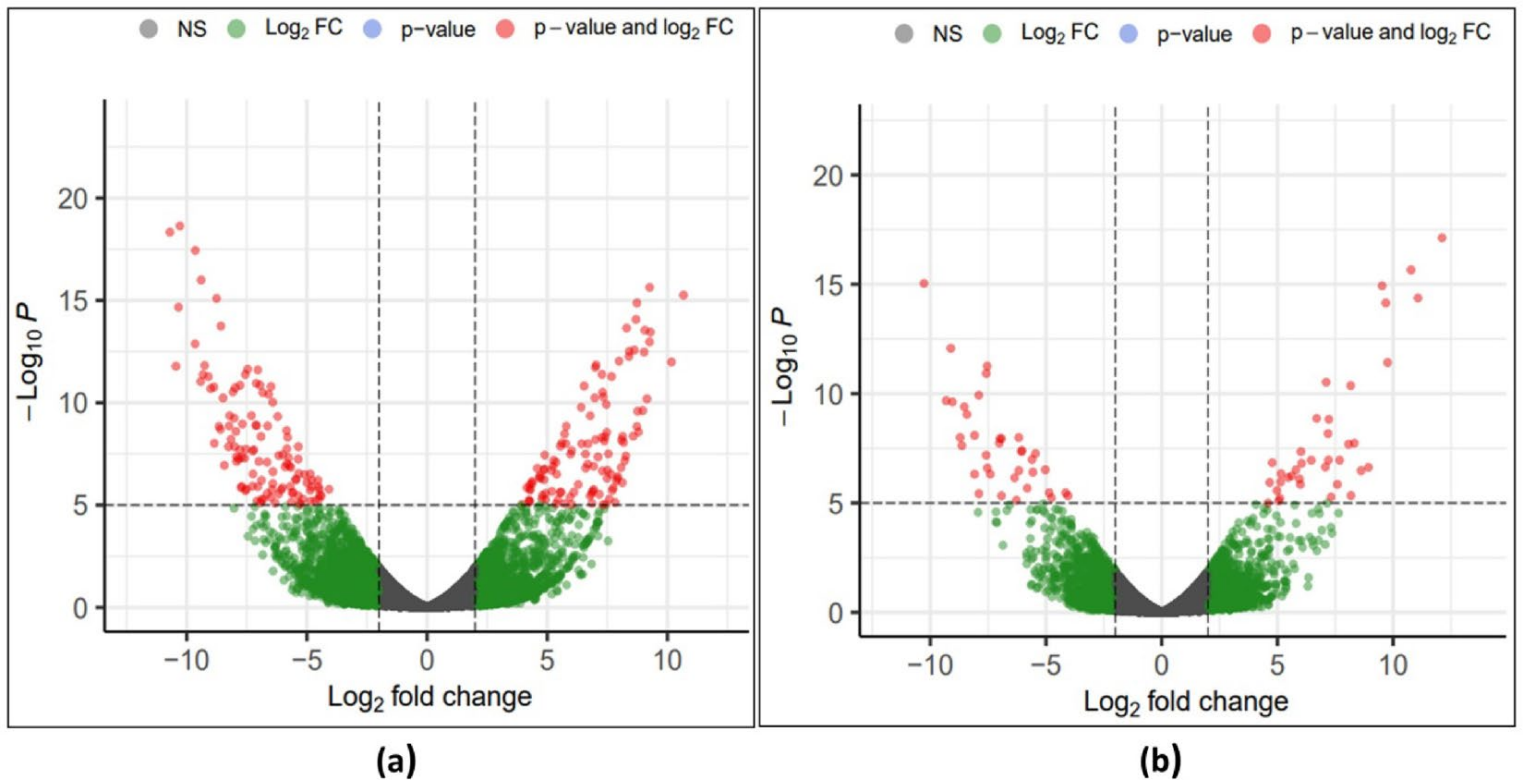
Comparison of differentially expressed genes (DEGs) in the leaf tissues between heat-tolerant and heat-sensitive genotypes at **(a)** flowering stage and **(b)** pod development stage.

Expression analysis was performed for the four samples, namely HTF (tolerant genotype at flowering stage), HTP (tolerant genotype at pod development stage), HSF (sensitive genotype at flowering stage), and HSP (sensitive genotype at pod development stage). Venn diagram analysis showed that 30,351 genes were common across the four samples (Fig. 4). The unique genes expressed in tolerant and sensitive genotypes at both stages were HTF (631 genes); HTP (674 genes); HSF (889 genes) and HSP (282 genes). The expression profile of the tolerant genotype was compared to the sensitive genotype at flowering (HTF vs. HSF) and pod development (HTP vs. HSP) stages respectively. A total of 2,467 DEGs were identified in both comparisons after adjusting the P value (< 0.05) and log2 fold change (> 2-upregulated; <−2-downregulated) (Supplementary Table S4). At the flowering stage (HTF_HSF), a total of 1631 DEGs were identified between the contrasting genotypes with 975 up-regulated and 656 down-regulated genes. While, at the pod development stage, (HTF_HSF), a total of 836 DEGs were identified, of which 477 genes were up-regulated and 359 were down-regulated (Fig. 5). Of the total 2,467 DEGs identified at both stages, 336 DEGs were commonly expressed (163 upregulated/156 down-regulated/17 altered regulation), 1295 DEGs (798 upregulated/497 down-regulated) were uniquely expressed at the flowering stage, and 500 DEGs (311 up-regulated/189 down-regulated) were uniquely expressed at the pod development stage.

### Functional annotation

The GO terms were assigned to the identified DEGs, and were annotated to cellular, biological, and molecular functions. The GO terms were assigned to the identified 2467 DEGs, of which 1076 sequences were annotated to 43 biological processes, 12 cellular components, and 37 molecular functions (Supplementary Table S5). At flowering and pod development stages, the cellular components mostly represented are an integral component nucleus (GO: 0005634), and cytoplasm (GO: 0005737). In the biological process, transmembrane transport (GO: 0055085), and carbohydrate metabolic process (GO: 0005975) were enriched and in the molecular functions, heme-binding (GO: 0020037), iron ion binding (GO: 0005506), monooxygenase activity (GO: 0004497) and oxidoreductase activity (GO: 0016491) were represented mostly.

**Fig. 6:**
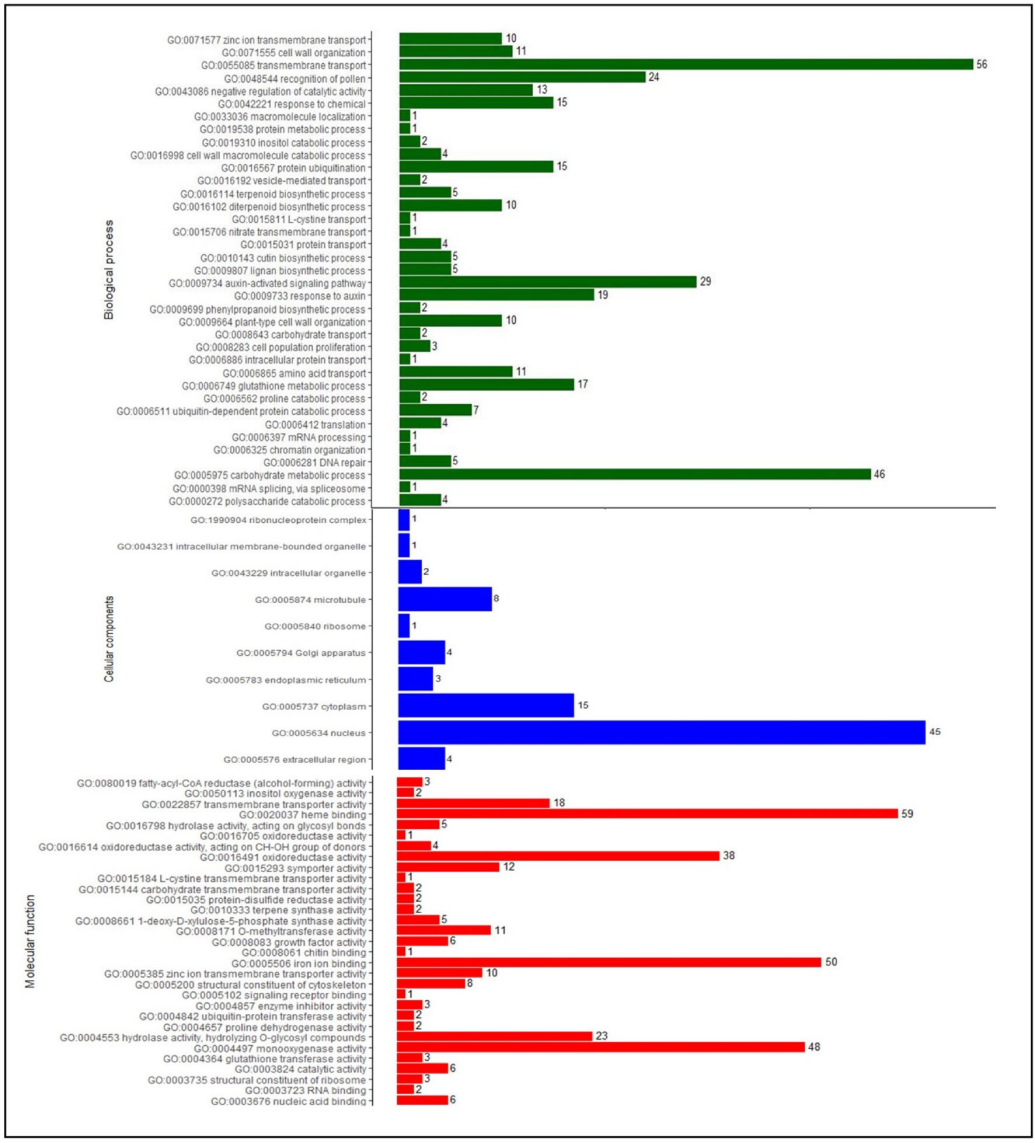
Functional annotations of DEGs under high-temperature stress at flowering stage. X-axis represents the DEGs count, Y-axis represents the Molecular function/Cellular components/Biological process.

At the flowering stage, 773 DEGs were annotated to cellular, biological, and molecular functions of which a total of 350 DEGs for the 37 biological processes, 339 DEGs for 31 molecular functions and 84 DEGs were annotated for 10 cellular components (Fig. 6). In the biological process, zinc ion transmembrane transport (GO: 0071577), macromolecule localization (GO: 0033036), protein metabolic process (GO: 0019538), cutin biosynthetic process (GO: 0010143), auxin response (GO: 0009733), phenylpropanoid biosynthetic process (GO: 0009699) and mRNA splicing via spliceosome (GO: 0000398) and in case of molecular function, zinc ion transmembrane transporter activity (GO: 0005385) and ubiquitin-protein transferase activity (GO:0004842) were unique to flowering stage.

**Fig. 7:**
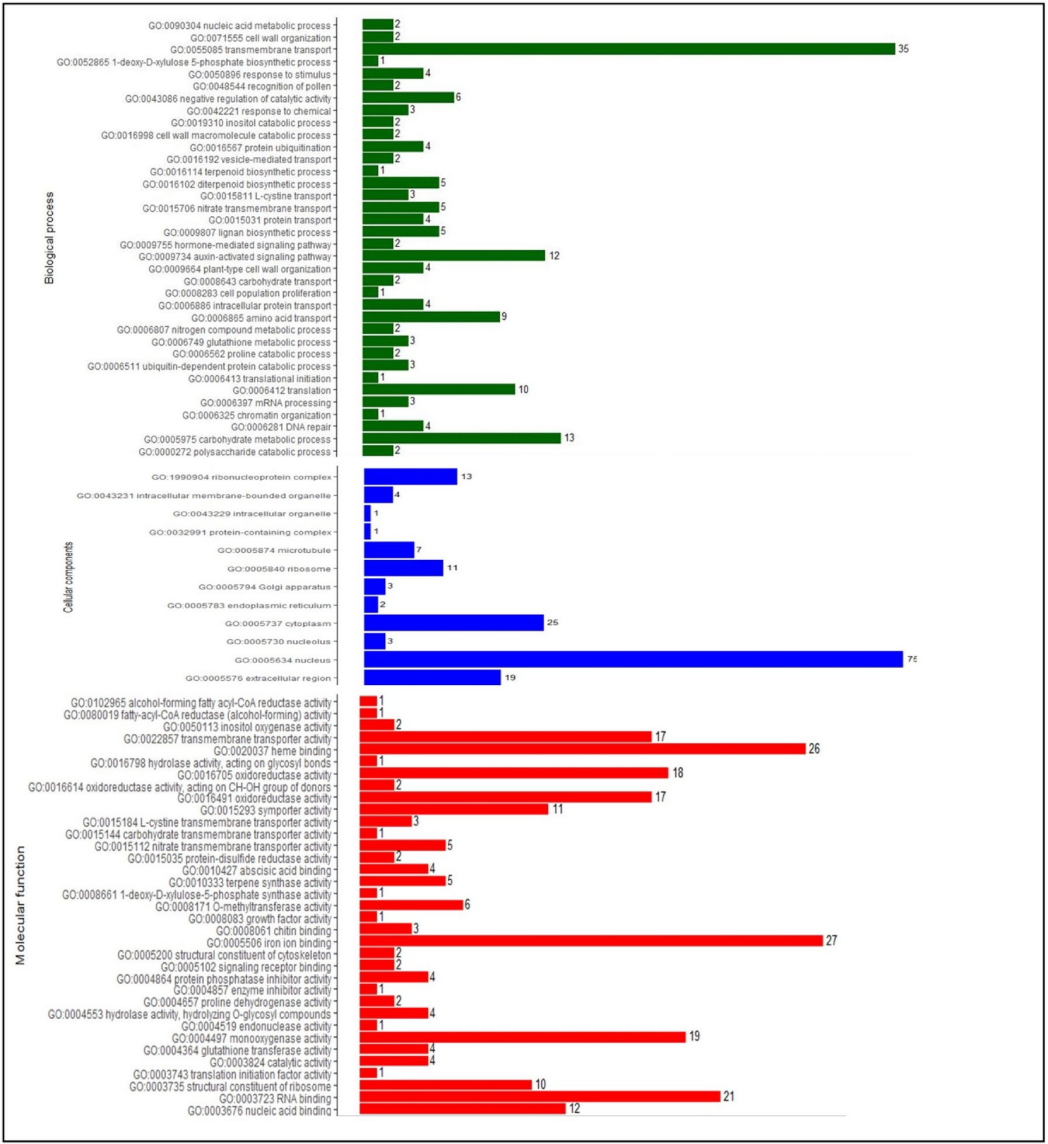
Functional annotations of DEGs under high-temperature stress at pod development stage. X-axis represents the DEGs count, Y-axis represents the Molecular function/Cellular components/Biological process.

At the pod development stage, 571 DEGs were annotated to cellular, biological, and molecular functions of which a total of 166 genes for the 36 biological processes, 241 genes for 35 molecular functions and 164 genes were annotated for 12 cellular components (Fig. 7). In the biological process, 1-deoxy-D-xylulose 5-phosphate biosynthetic process (GO: 0052865), response to stimulus (GO: 0050896), hormone-mediated signaling pathway (GO: 0009755), nitrogen compound metabolic process (GO: 0006807), translational initiation (GO: 0006413) and in case of cellular components, protein-containing complex (GO: 0032991) and nucleolus (GO: 0005730) were unique to pod stage. Among the molecular functions, alcohol-forming fatty acyl-CoA reductase activity (GO: 0102965), nitrate transmembrane transporter activity (GO: 0015112), abscisic acid binding (GO: 0010427), protein phosphatase inhibitor activity (GO: 0004864), endonuclease activity (GO: 0004519) and translation initiation factor activity (GO: 0003743) were unique to only pod stage.

**Fig. 8:**
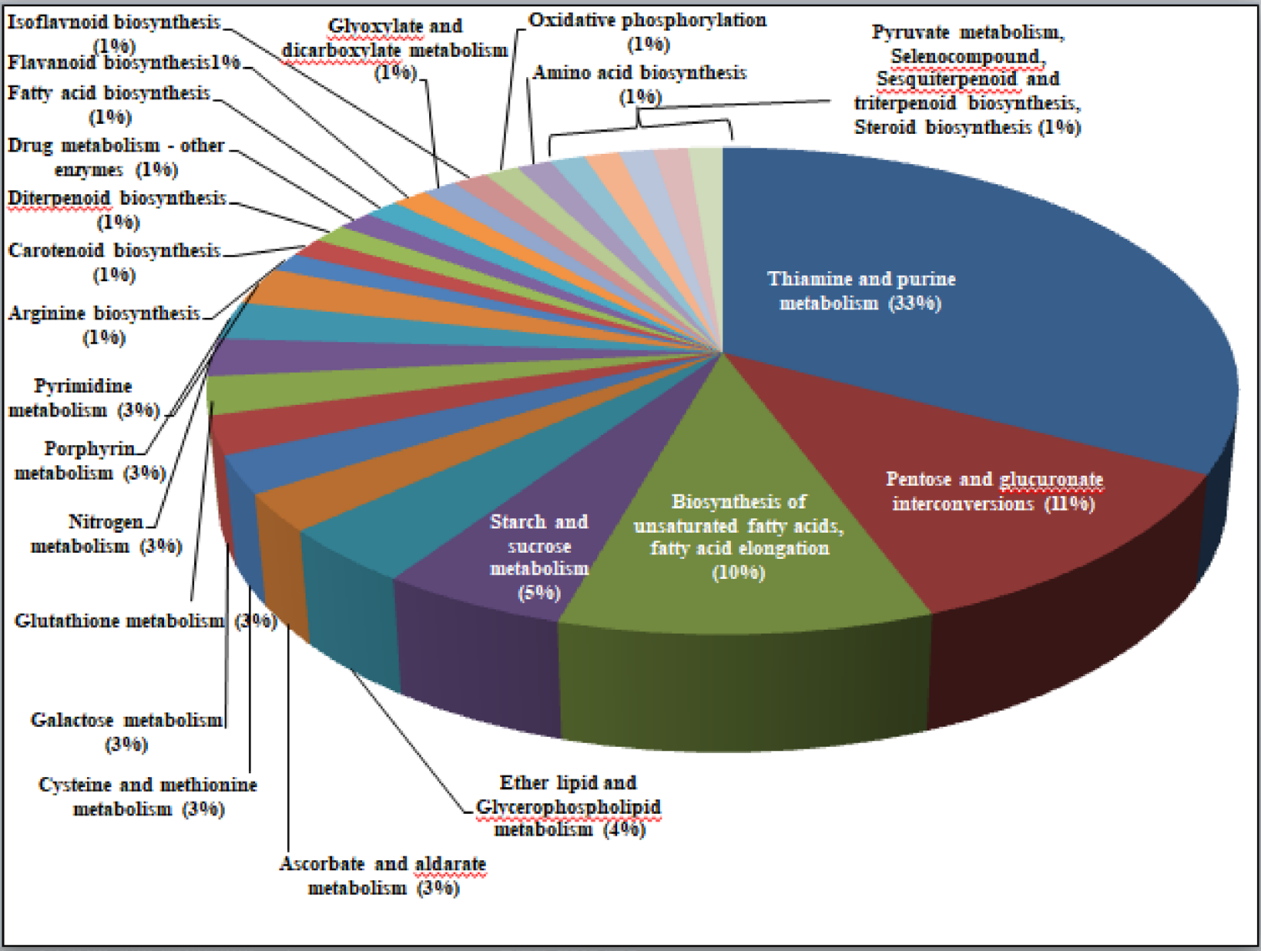
Pie chart denoting the distribution of pathways to the identified DEGs at flowering stage under high-temperature stress conditions.

**Fig. 9:**
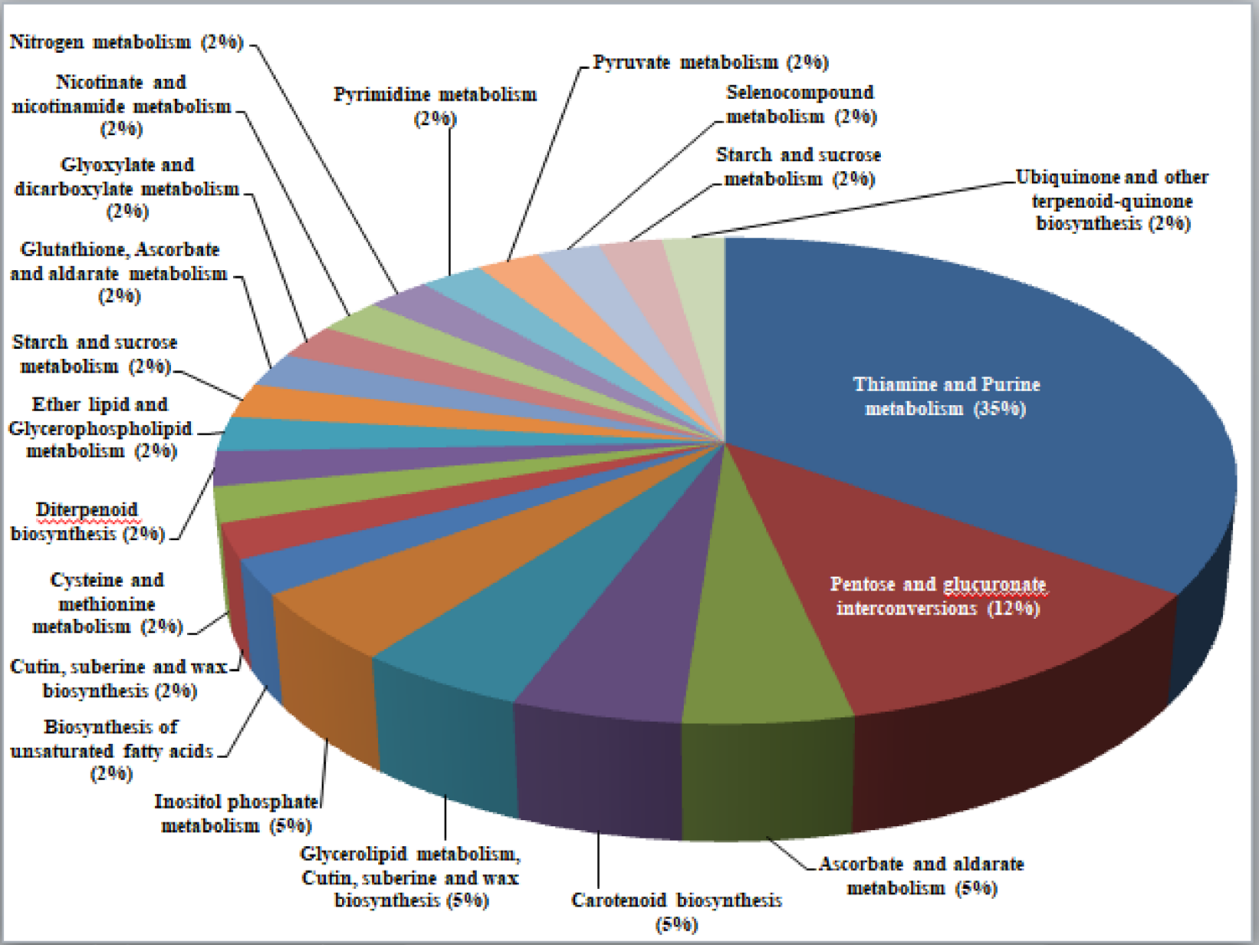
Pie chart denoting the distribution of pathways to the identified DEGs at pod development stage under high-temperature stress conditions.

Besides GO terms, KEGG pathway analysis was used to assign biological pathways to the identified DEGs. A total of 106 DEGs were assigned to 46 pathways (Supplementary Table S6). Around 22 pathways were common at both stages. At the flowering and pod development stages, most of the DEGs were involved in thiamine and purine metabolism, pentose and glucouronate interconversions, and starch and sucrose metabolism pathways accounted for > 50%. Less number of DEGs are involved in pyruvate metabolism and oxidative phosphorylation. The enriched pathways at the flowering and pod development stages were shown in Figs. 8 and 9. A total of 17 and 7 pathways were unique to the flowering and pod development stages, respectively.

### High-Temperature stress responsive genes

To gain a deeper understanding of the major categories of genes differentially expressed under high temperature stress, we conducted a thorough review of the literature for all the differentially expressed genes (DEGs). Based on the literature review, sixteen stress-responsive genes were identified from the current study. The identified stress-responsive genes include *Ribulose bisphosphate carboxylase small chain 1*,* chloroplastic* (2.20 fold change, FC); *inositol-3-phosphate synthase* (2.26 FC); *LRR receptor-like serine/threonine-protein kinase* (3.27 FC); *GDSL esterase/lipase APG* (2.14 FC); *inositol oxygenase 2* (4.08 FC); *malate dehydrogenase [NADP]*, *chloroplastic* (7.94 FC); *NAD-dependent malic enzyme 2* (2.67 FC); *Pentatricopeptide repeat-containing protein* (3.14 FC); *sucrose synthase 2* (2.32 FC); *1-acyl-sn-glycerol-3-phosphate acyltransferase-like* (2.48 FC); *ras-related protein Rab7* (4.61 FC); *AAA-type ATPase* (3.28 FC); *glucose-6-phosphate 1-dehydrogenase 3* (3.57 FC); *nicotinate phosphoribosyl transferase 1* (2.67 FC); *glutamate synthase [NADH]* (2.76 FC) and *ATP-dependent zinc metalloprotease FTSH 12*,* chloroplastic* (2.87 FC). These genes play an important role in processes such as photosynthetic activity, reducing transpiration loss, antioxidant activity and nucleotide and carbohydrate metabolism. Higher expression of these genes during the flowering or pod development stages contributed greater adaptability in the tolerant genotype compared to the sensitive genotype. A list of the selected high-temperature stress responsive genes and their corresponding primer sequences are provided in Table 2.

### Validation of the differentially expressed genes by qPCR

**Fig. 10:**
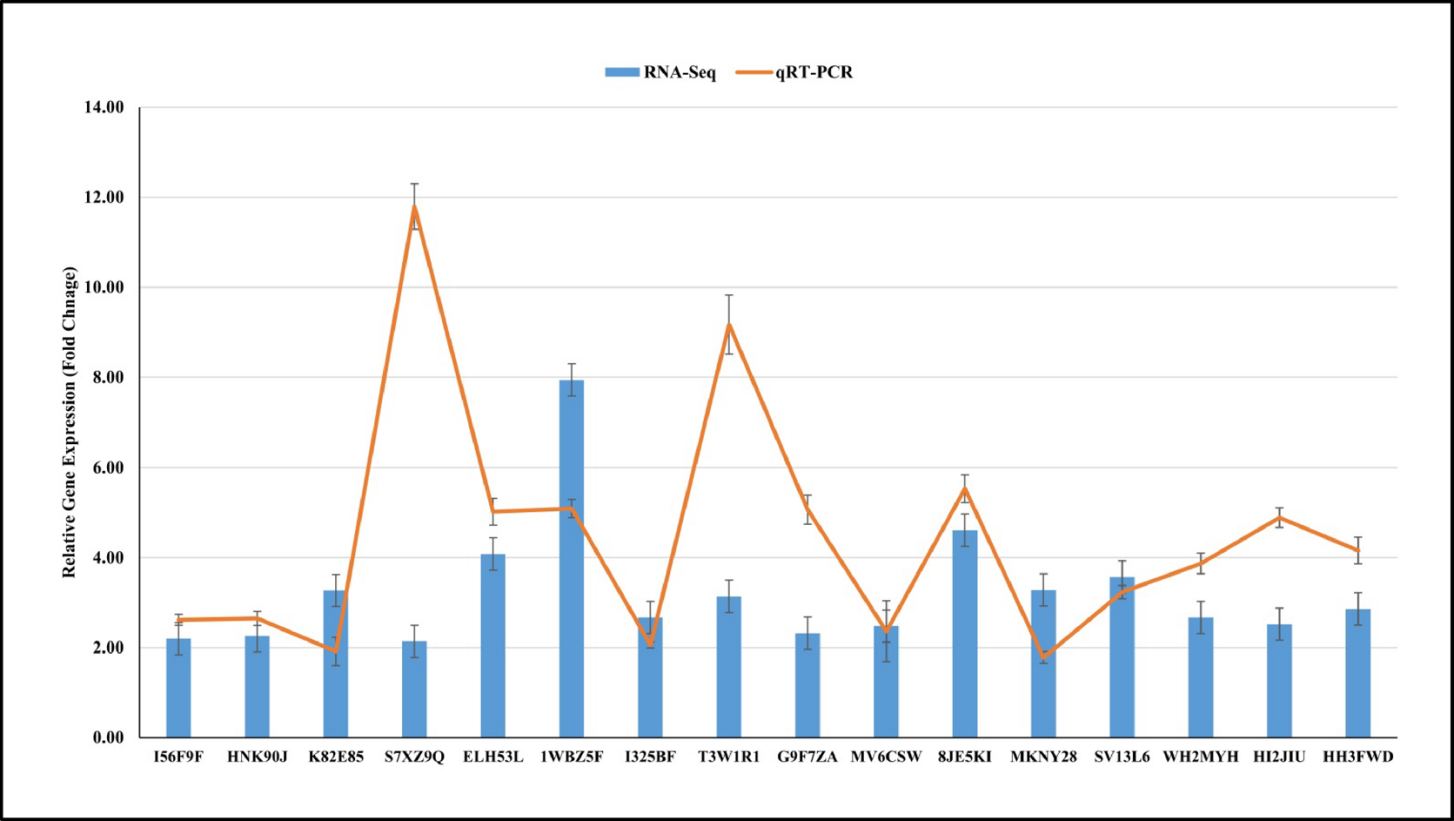
Relative gene expression patterns of selected high-temperature stress responsive genes analyzed by qRT-PCR. X-axis represents the selected stress responsive genes; Y-axis represents the relative gene expression in fold change values.

After qPCR analysis, mean values of replicates are obtained, and C_t_ values of samples are determined. Sample C_t_ values were normalized by comparing them with C_t_ values of housekeeping gene. Relative fold change values are calculated by using the 2^−^^Ct^ method. All the selected genes reported similar expression patterns in the HT stress tolerant genotype compared to the sensitive genotype, which were reported by RNA-Seq (Fig. 10). A positive correlation between the log2fold change values of qRT-PCR and RNA-Seq was observed (Pearson correlation *R* = 0.14) (Supplementary Fig. S2).

## Discussion

Rise in temperature above a threshold limit for a crop is detrimental to its growth and development^36^. In groundnut, studies reported that a temperature rise of > 35℃ would decrease the pod yield up to 43%^11^ and 15–25%^12^. The reproductive stage in groundnut is sensitive to high temperatures, resulting in flower abortion, poor seed filling, and thus reduced pod yield^10^. Based on the agronomic performance of 64 genotypes screened across eight HT stress environments, ICGV 16553 and ICGV 16516 were identified as heat-tolerant and -sensitive genotypes, respectively. To understand physiological, biochemical, and molecular mechanisms underlying HT stress tolerance in groundnut, the contrasting genotypes were evaluated for total antioxidant activity (TAA), total phenol content (TPC), and translocation of assimilates from source to sink. Comparative transcriptomic analysis was conducted and selected DEGs at flowering and pod development stages were validated. The plants were raised in growth chamber for HT stress experiments that used temperature regime based on the daily and monthly temperatures recorded during summer 2019 & 2020.

### Total antioxidant activity & total phenol content for high temperature stress

Under HT stress conditions, reactive oxygen species (ROS) accumulation will lead to lipid peroxidation in cell membranes, altered cellular metabolism, nicking of DNA, protein oxidation and destruction of pigments^23^. To combat ROS production, tolerant plants induce an antioxidant system that acts as a ROS scavenging agent^37^. We have observed that TAA and TPC are different at flowering and pod development stages, suggesting stage-specific responses to HT stress conditions in tolerant and sensitive genotypes. At flowering stage, TAA and TPC increased in tolerant genotype (ICGV 16553) suggesting the role of increased antioxidant capacity in HT stress tolerance. On contrary, TAA and TPC declined in sensitive genotype (ICGV 16516) in response to HT stress compared to the control conditions. Higher antioxidant capacity was observed in the tolerant genotypes of lentil via ascorbate-glutathione pathway under HT stress conditions^13^. Under HT stress, it was observed that the enzymatic antioxidants (ascorbate peroxidase, glutathione reductase) and non-enzymatic antioxidants (ascorbic acid) activity increased; however, glutathione decreased in the tolerant mungbean genotype^21^. While HT stress declined TAA and TPC of both the tolerant and sensitive genotypes at the pod development stage, indicating the sensitivity of both the genotypes. However, further research regarding the stage-specific responses of the antioxidant system is required in groundnut.

### Carbon partitioning pathways for High-Temperature stress resilience

Carbon partitioning in legumes involves the distribution of carbon fixed during photosynthesis to various tissues and metabolic pathways, which is essential for growth and storage. HT stress causes leaf senescence, inhibits net photosynthetic rate, and disrupts sucrose-starch conversion and loss of sink activity, thus affecting the seed weight and quality^38^. Earlier studies focused on harvest index, crop growth rate, pod growth rate and partitioning factor to understand the accumulation and partitioning of photosynthates in groundnut^11,39^.

In this study, we have attempted to study the carbon partitioning in groundnut with the sugar profiling and identification of candidate gene and associated pathways at reproductive stage. This study revealed that the tolerant genotype (ICGV 16553) recorded increased glucose and sucrose content in the leaves at the flowering stage under HT stress compared to the control conditions. The increased sugar content in the leaf tissue acts as osmo-protectants and energy sources imparting tolerance to drought/heat stress^40^. While, at the pod stage, we observed increased glucose levels and reduced sucrose levels in the leaves and pod samples, respectively. The high levels of glucose in the pods are indicative of an active sucrose metabolism under HT stress and it is most likely directed to the synthesis of starch. Sucrose import and supply are affected by cell-wall bound invertase enzyme that degrades sucrose to hexoses^41^. In another study, tolerant genotype, ICGS 44 showed > 60% increase in glucose content and comparatively low reduction in sucrose content over the sensitive groundnut genotypes under high HT stress^12^. Also, the accumulation of starch in the harvested kernels showed that similar amount of starch was accumulated in tolerant genotype under control and HT stress conditions, indicating effective translocation of assimilate from source to sink under HT stress conditions. Efficient carbon translocation into pods and/or to kernels and high starch accumulation resulted in HT tolerance of the tolerant genotype.

In sensitive genotype (ICGV 16516), both glucose and sucrose decreased in the leaves at flowering stage and in both leaves and pods at pod development stage under HT stress compared to the control conditions. The reduced accumulation of sugars in the leaves was attributed to the impairment of photosynthesis during HT stress^42^. Moreover, the sucrose accumulated in the leaves was poorly translocated to the pods in sensitive genotype, leading to reduced glucose levels in pods under HT stress conditions. This is attributed to the inhibited sucrose metabolism due to loss of invertase activity in leaves and impaired sucrose supply to developing seeds, also reported in chickpea^20^ and mungbean^24^. The starch accumulation in ICGV 16516 declined by 38%, due to the altered expression of starch-related genes under HT stress^43^ that leads to reduction in yield^44^. In this study, the sensitive genotype accumulated 38% less starch in pods under HT stress suggesting possible alteration in starch-related genes. Poor carbon translocation occurred from leaves to pods due to impaired sucrose metabolism and sucrose supply in sensitive genotype under HT stress.

The primary pathway for carbon fixation is the Calvin cycle, where the RuBisCO enzyme captures CO₂ and converts it into 3-carbon compounds (glyceraldehyde-3-phosphate), which are then used to produce glucose and sucrose. Glucose is either stored as starch in chloroplasts, particularly in seeds, or converted into sucrose, which is transported through the phloem to different plant parts. These processes are regulated by enzymes such as ADP-glucose pyrophosphorylase (AGPase) and sucrose synthase. Deciphering mechanism also requires an understanding of the molecular basis of HT stress tolerance. In the present study, the gene encoding *sucrose synthase 2* (*arahy.*G9F7ZA) encodes a reversible enzyme that converts sucrose to glucose and fructose. Sucrose synthase may also play a role in starch and sucrose metabolism pathway under HT stress^45^. The carbon partitioning studies revealed that glucose levels were high in tolerant genotype at pod development stage in the leaves, and our transcriptome study also revealed the upregulation of *sucrose synthase 2* at the pod stage, thus confirming its role under HT stress in groundnut. Additionally, carbon is utilized in cellular respiration within mitochondria, where ATP is generated via the pyruvate dehydrogenase enzyme. Some carbon is also diverted into secondary metabolism, producing compounds such as flavonoids and alkaloids, which play key roles in plant defense and signaling^46^. In the present study, carbon partitioning pathways, including biosynthesis of unsaturated fatty acids, fatty acid elongation; ether lipid metabolism, glycerophospholipid metabolism; galactose metabolism, starch and sucrose metabolism; glyoxylate and dicarboxylate metabolism; carbon fixation in photosynthetic organisms, pyruvate metabolism, glycolysis/gluconeogenesis, and the citrate cycle (TCA cycle), were enriched during both the flowering and pod development stages. The DEGs involved in these pathways were upregulated, resulting in efficient fixation of carbon and their partitioning to various tissues, which leads to increased tolerance in HT genotype.

### High-Temperature stress responsive genes

In recent years, RNA sequencing (RNA-seq) has become an important technique to investigate the molecular response to stress conditions and researchers have successfully employed RNA-seq to decipher the mechanism of HT stress responses in crop species, such as, soybean^29,30,47^, lentil^32^, pigeon pea^33^ and common bean^31^. Transcriptome profiling of groundnut was carried out to understand the mechanisms for tolerance to drought^34,35^. In the present study, the gene expression of the contrasting genotypes was studied to identify the HT stress specific genes at flowering and pod development stages in groundnut. The number of up-regulated DEGs was higher than the down-regulated DEGs at both flowering and pod development stages, illustrating dynamic transcriptional changes in tolerant genotype.

The identified DEGs during the flowering and pod development stages related to HT stress in groundnut are given in Table 2. During the flowering stage, genes encoding *Inositol-3-phosphate synthase* (*arahy.*HNK90J) and *Ribulose bisphosphate carboxylase small chain 1* (*arahy.I56F9F*), involved in inositol phosphate metabolism and glyoxylate and dicarboxylate metabolism pathways, were upregulated and reported to have high photosynthetic efficiency during elevated temperature stress, also reported in wheat^48^ and rice^49^. These findings agree with high yield performance and high SPAD chlorophyll content of tolerant genotype (ICGV 16553) identified based on multi-season data across environments (Unpublished data). *LRR receptor-like serine/threonine-protein kinase* (*arahy.*K82E85) gene belongs to the largest subfamily of receptor-like kinases (RLK) and involved in the pyrimidine metabolism pathway, plays critical role in plant development and stress responses. In Arabidopsis, overexpression of the *AtRPK1* gene lead to decreased transpiration loss and consequently increased tolerance to drought stress^50^.

At the pod development stage, genes encoding *inositol oxygenase 2* (*arahy.*ELH53L) involved in ascorbate and aldarate metabolism pathway, *NAD-dependent malic enzyme 2* (*arahy.*I325BF) and *Malate dehydrogenase [NADP]* (*arahy.*1WBZ5F) involved in pyruvate metabolism pathway, *GDSL esterase/lipase APG* (*arahy.*S7XZ9Q) and *Glucose-6-phosphate 1-dehydrogenase* 3 (*arahy.*SV13L6) involved in Thiamine and Purine metabolism pathways and *Nicotinate phosphoribosyl transferase 1* (*arahy.*WH2MYH) involved in nicotinate and nicotinamide metabolism pathway were upregulated and they were found to be involved in the anti-oxidative defense system^48,51–54^. However, in our study, antioxidant activity decreased at pod stage in both tolerant and sensitive genotypes. Further studies are needed to understand the stage specific genotype responses to HT stress in groundnut. *Pentatricopeptide repeat-containing protein* (*arahy.*T3W1R1) genes belongs to a large family of eukaryotic RNA-binding proteins that played key role in abiotic stress. In rice, a total of 75 pentatricopeptide (PPR) genes were up-regulated under salt stress conditions and 73 PPR genes were up-regulated under the drought stress conditions compared with the control, thus confirming their overexpression under abiotic stress conditions^55^. It was reported that genes encoding *pentatricopeptide repeat-containing protein* and *Malate dehydrogenase* were underlying in Cluster-2-Ah12 hotspot genomic region present on QTL Cluster-2 was observed during the seed development stage in groundnut under HT stress conditions^28^. A gene encoding for *heat shock 70 kDa protein* (*arahy.*I6A5S7) was upregulated during pod stage in tolerant genotype (ICGV 16553). In earlier study, it was reported that tolerant genotype (ICGS 44) showed sharp increase in small (HSP 17 & HSP 90) and larger molecular weight HSPs (HSP 70 & HSP 90) when exposed to HT stress (45°C) compared to the sensitive groundnut genotypes^12^. Such rapid and long duration induction of HSPs prevents HT stress injuries in tolerant genotype.

The gene *1-acyl-sn-glycerol-3-phosphate acyltransferase* (*arahy.*MV6CSW) was expressed during pod stage and contributes to membrane stabilization under stress responses in maize^30^. The gene *ras-related protein Rab7* (*arahy.*8JE5KI) is involved in membrane trafficking, protein secretion and regulation of exocytosis^56^. *AAA-type ATPase*(*arahy*.MKNY28) gene was upregulated during pod stage and this gene encodes for numerous cellular activities, seed maturation process during salinity stress^57^.

At both flowering and pod development stages, the gene *glutamate synthase [NADH]* (*arahy.HI2JIU*) having a role in amino-acid metabolism pathway was upregulated and inhibits chlorophyll degradation and activates amino acid metabolism involved in energy production, antioxidant activity, and nitrogen balance^58^. *ATP-dependent zinc metalloprotease FTSH 12 (arahy.*HH3FWD*)* involved in Thiamine and Purine metabolism pathways plays a key role in chloroplast development and plant response to stress. The above discussed DEGs at both flowering and pod development stages were further validated using qPCR. These findings suggest that the genes identified through RNA-Seq analysis have potential applications in enhancing groundnut crop tolerance to HT stress.

## Methods

### Plant material

Two Spanish Bunch groundnut genotypes namely, ICGV 16553 (heat-tolerant) and ICGV 16516 (heat-sensitive) were selected based on the field evaluation at International Crops Research Institute for the Semi-Arid Tropics (ICRISAT), Patancheru and Dr. Panjabrao Deshmukh Krishi Vidyapeeth (PDKV), Akola, Maharashtra during the summer seasons of 2020 and 2021 for agronomic performance under HT stress conditions. The heat-tolerant genotype was identified based on high mean pod yield, high stability, high SPAD chlorophyll meter reading (SCMR), and good recovery for large kernel grade. The cultivar, 55–437 is a HT stress-tolerant cultivar grown in Sahelian zone of West Africa^15,39^ and was one of the parents of the HT tolerant genotype, ICGV 16553. Conversely, the heat-sensitive genotype was identified by its lower pod yield, low SCMR, and poor recovery for large kernel grade under similar stress conditions (Unpublished data).

### High-temperature stress treatment

The experiment was conducted in a controlled growth chamber facility (Conviron, Model PG-36, Winnipeg, MB, Canada) at ICRISAT, Hyderabad, India. The height of the growth chamber is 2.0 m and the area is 3.3 m^2^ with a touch screen unit for programming and data-logging features. The photosynthetic active radiation (PAR) was 1370 µmol/m^2^sec and CO_2_ was maintained at around 380 ppm during the experimental period.

Seeds were sown in two replications and each replication consisted of three plants per pot. Each pot is about 30 cm in diameter and filled with an autoclaved mixture of sand, red soil, and farm yard manure (FYM) in a 3:2:1 ratio. The control treatment was kept outside the growth chamber, and the other treatment in the growth chamber was exposed to high temperatures (40°C) for about two hours (13.00–15.00 h.) at flowering and pod development stages. The temperature regime for the experimental duration was based on the daily and monthly temperatures recorded during the summer 2019 and 2020 seasons at ICRISAT, Hyderabad, India. The plants were grown in 12-hour day and 12-hour night cycles and humidity of 40/60% with continuous monitoring at intervals throughout the experimental period. Optimum irrigation was provided based on the requirement.

### Estimation of total antioxidant activity (TAA) and total phenol content (TPC)

TAA of the sample was determined using the ferric reducing antioxidant power (FRAP) assay^59^. A potential antioxidant will reduce the ferric ion (Fe^3+^) to the ferrous ion (Fe^2+^); the latter forms a blue complex (Fe^2+^/TPTZ). The blue-colored complex developed was read at 650 nm in a UV spectrophotometer.

Phenols were estimated by the Folin-Ciocalteu (FC) method^60^. It is based on the reaction between phenols and an oxidizing agent, phospho-molybdate which results in the formation of a blue-colored complex at 650 nm. Leaf sampling was carried out at flowering (35 days after sowing - DAS) and pod development (65 DAS) stages to determine the total antioxidant activity and total phenolic content.

### Estimation of sugars and starch content

Sucrose and glucose were estimated from leaf and pod samples using High-Performance Liquid Chromatography (HPLC). Sampling was carried out at the flowering (leaf tissue) – 35 DAS and pod development stage (leaf and pod tissues) – 65 DAS for estimation of sugar content. Starch content was estimated using an anthrone reagent (Sigma-Aldrich), following the procedures^61,62^. The starch content was estimated from the kernels and is reported as a milligram of soluble glucose.

### RNA extraction and cDNA library Preparation

Leaf sampling was done from two contrasting genotypes, ICGV 16553 (tolerant) and ICGV 16516 (sensitive) at both stages (flowering − 35 DAS and pod development − 65 DAS), namely HTF (tolerant genotype at flowering stage), HTP (tolerant genotype at pod development stage), HSF (sensitive genotype at flowering stage), and HSP (sensitive genotype at pod development stage). Five randomly selected leaves from both the genotypes were sampled and pooled individually, at both flowering and pod development stages. Leaf samples were chosen for RNA sequencing as they are the primary sites for transpiration and photosynthesis, making them highly responsive to heat stress. Additionally, leaves often exhibit more pronounced gene expression changes under stress conditions, providing valuable insights into the plant stress response. RNA extraction was done using the NucleoSpin RNA Plant kit (Macherey-Nagel), according to the manufacturer’s instructions. The quality and quantity of RNA were evaluated by using Qubit 2.0 fluorometer (Life Technologies, Inc). cDNA libraries were synthesized from the extracted RNA samples pooling in equal ratios and used for 2 × 126-bp paired-end sequencing on a single lane of the Illumina Hi Seq 2500 (Illumina Inc., San Diego, CA, USA). Illumina clusters were generated and loaded onto the Illumina Flow Cell on the Illumina Hi Seq 2500 sequencer.

### Functional annotation

The raw data for each sample were checked for quality using FastQC v0.11.6 (https://www.bioinformatics.babraham.ac.uk/projects/fastqc/). Low-quality reads (< Q20) and adapter sequences were filtered through Trimmomatic v0.39^63^. The filtered reads were aligned to the cultivated groundnut (*Arachis hypogaea* L.) reference genome (cultivar Tifrunner) version gnm2 from PeanutBase (https://www.peanutbase.org/) using HiSat2 v2.2.1. The read counts for each gene feature in each sample were estimated using feature Counts from subread package v. 2.0.3. A gene feature was considered to be expressed when read count ≥ 10 in at least one sample, and was said to be significantly differentially expressed when |log2(fold change)| ≥ 2 and P-value < 0.05. The genes were functionally annotated against NCBI’s non-redundant (nr) protein database (taxon. Viridiplantae) using standalone blast (e-value ≤ 1E − 5) followed by Gene Ontology (GO) and KEGG pathway analysis using Blast2GO v5.2. The workflow is depicted in the Supplementary Fig. S1.

### Validation of the differentially expressed genes (DEGs) by qPCR

Real-Time PCR (qPCR) was conducted to validate the expression patterns of randomly selected DEGs for HT stress tolerance, identified by RNA-Seq. Synthesized cDNA was subjected to qPCR, by using SYBR Green Master Mix, according to the manufacturer’s instructions. The experiment was conducted with three biological replicates per sample with qPCR reagents, such as synthesized cDNA, SYBR Green Master Mix, forward and reverse primers, and nuclease free water (NFW). adh-3 gene was used as internal control or reference gene due to its expression across various tissues, developmental stages, and stress conditions^64^. Its consistent role in maintaining cellular integrity makes it suitable for accurate normalization in gene expression analysis. The thermal profile for qPCR includes an initial denaturation at 95°C for 3 min, followed by 40 cycles of denaturation at 95°C for 10 s, annealing at 60°C for 30 s and extension at 72°C for 10 s. The final extension was performed at 72°C for 10 s. The qPCR primers were designed by using PrimerQuest™ bioinformatics tool (https://www.idtdna.com/pages/tools/primerquest). A relative quantification assay has been conducted in Applied Biosystems^®^ 7500 Real-Time PCR system (Applied Biosystems). Threshold cycle (C_t_) values were determined, and fold changes (FC) were calculated by using the 2^−^^Ct^ method^65^.

## Conclusion

High-temperature stress tolerance is important for sustaining production in future climate change scenarios where the temperatures are on a rise. The heat-tolerant and heat-sensitive genotypes identified based on agronomic performance in the field under HT stress showed significant differences in several biochemical constituents. Accumulation of antioxidants and phenols in the leaves at the flowering stage is an indication of effective defense mechanism in the tolerant genotype, ICGV 16553 under HT stress. Efficient carbon partitioning in the tolerant genotype indicated by increased glucose levels in the pods suggests active sucrose metabolism associated with high starch accumulation that contributes for yield stability under HT stress. Thereby, the tolerant genotype exhibited better physiological and metabolic conditions under HT stress. The identified DEGs encoded genes related to photosynthetic efficiency, heat shock response, sucrose metabolism, transcriptional activity, and antioxidant system. Future studies must focus on enzyme activity, sugar transporters and their regulation which are involved underneath sucrose metabolism and utilization of the identified candidate genes governing HT stress tolerance in groundnut. The understanding of mechanisms underlying HT stress tolerance and identification of candidate genes enables genetic enhancement for HT stress tolerance in groundnut.

**Table 1:** Summary of the RNA-Seq data generated at flowering and pod development stages in contrasting groundnut genotypes under high-temperature stress conditions.

Sample details	Stage of sampling	Raw Bases (In Gb)	Raw Reads (In Millions)	Filtered Bases (In Gb)	Filtered Reads (In Millions)	Hisat2 alignment rate (%)
Heat-tolerant genotype (ICGV 16553)	Flowering stage	4.79	38.81	4.71	38.39	98.61%
Heat-sensitive genotype (ICGV 16516)		4.84	39.23	4.75	38.78	98.73%
Heat-tolerant genotype (ICGV 16553)	Pod development stage	5.14	41.76	5.06	41.28	98.72%
Heat-sensitive genotype (ICGV 16516)		3.93	32.11	3.85	31.49	98.47%
Total		18.7	151.91	18.37	149.9	98.60%

**Table 2:** High-temperature stress responsive genes and their respective primer sequences, selected for validation by qRT-PCR.

S.no	Gene description	Gene ID	Primer	Primer Sequence
1	Ribulose bisphosphate carboxylase small chain 1, chloroplastic	arahy.I56F9F	F	CAAGCCAGAGGGCTACTAAAT
			R	GAAAGCAGAAGCAGAGTTGTTAC
2	Inositol-3-phosphate synthase	arahy.HNK90J	F	TGGAGAAGGTGGTTGTTCTATG
			R	GTTCCTGTCCAAGGCACTTA
3	LRR receptor-like serine/threonine-protein kinase	arahy.K82E85	F	CGGCCTCAATCTCAGCTATAA
			R	CTGAGAGCTTGTTGTGTGAAAG
4	GDSL esterase/lipase APG	arahy.S7XZ9Q	F	CACTTGTTCCGGCAATCATAAC
			R	CCTCCCATAAGGAGGGTAATTTG
5	Inositol oxygenase 2	arahy.ELH53L	F	TGAGCAACCTATCTTCGATTCAC
			R	CCATTCTGAGACACCTCCTTTG
6	Malate dehydrogenase [NADP], chloroplastic	arahy.1WBZ5F	F	CAGAGAGGTGGTGTGCTTATTC
			R	CCCTTGGGAGTAGGAGTGATTA
7	NAD-dependent malic enzyme 2	arahy.I325BF	F	CTGTTGTCTGTCTCCAGTTCTC
			R	TTCCTTCCCACGACCAAATAG
8	Pentatricopeptide repeat-containing protein	arahy.T3W1R1	F	CTCCCATACACTTCCACAAGAG
			R	GTCCCTGCAAACTCGGATTA
9	Sucrose synthase 2	arahy.G9F7ZA	F	AGAGTCTCTCCTCCAGGTTATC
			R	CCATTTCGCCTCTCACAACTA
10	1-acyl-sn-glycerol-3-phosphate acyltransferase-like	arahy.MV6CSW	F	GCCGAGAAGATCGATGACTATG
			R	GGTGTGGACTATGACTATGAACC
11	Ras-related protein Rab7	arahy.8JE5KI	F	GCCTGAGACTGTTCCAGATAAC
			R	TAGCCATGGCATTAAGAGGATAAA
12	AAA-type ATPase	arahy.MKNY28	F	GGGTGGCAGACCGAATAAA
			R	GGTCAAGAATGCTGGGAAGTA
13	Glucose-6-phosphate 1-dehydrogenase 3	arahy.SV13L6	F	GCTTCTAATCGCCTGTTCTATCT
			R	TGACTCTTGTCCAACCATTACC
14	Nicotinate phosphoribosyl transferase 1	arahy.WH2MYH	F	CCTCACCGATCTCTACCAATTC
			R	CGGATTCTTCCGGAAATACAAATC
15	Glutamate synthase [NADH]	arahy.HI2JIU	F	GGCTGATTATGGTCGCTTCT
			R	GCTTGTCGTCCCTCTGAAATA
16	ATP-dependent zinc metalloprotease FTSH 12, chloroplastic	arahy.HH3FWD	F	GAGAATGCACAAGCACACTTAC
			R	GAGCAAGCATAGGGAGAGATG

## Supplementary Information

Below is the link to the electronic supplementary material.

Supplementary Material 1

Supplementary Material 2

## Data Availability

RNA-seq data that support the findings of this study have been deposited in the National Biotechnology Center Archives database with the primary accession code PRJNA1197725. (http://www.ncbi.nlm.nih.gov/bioproject/1197725)
