# Supplementary material for: Transcriptome analysis of genes and carbon partitioning pathways involved in high temperature stress resilience in groundnut (Arachis hypogaea L.)
Source: Sci Rep. 2025 Aug 15;15:29939. doi: 10.1038/s41598-025-15509-4 (PMC12356953; doi:10.1038/s41598-025-15509-4)
Supplement: Supplementary file 1 — Supplementary Material 1 [file 41598_2025_15509_MOESM1_ESM.docx]

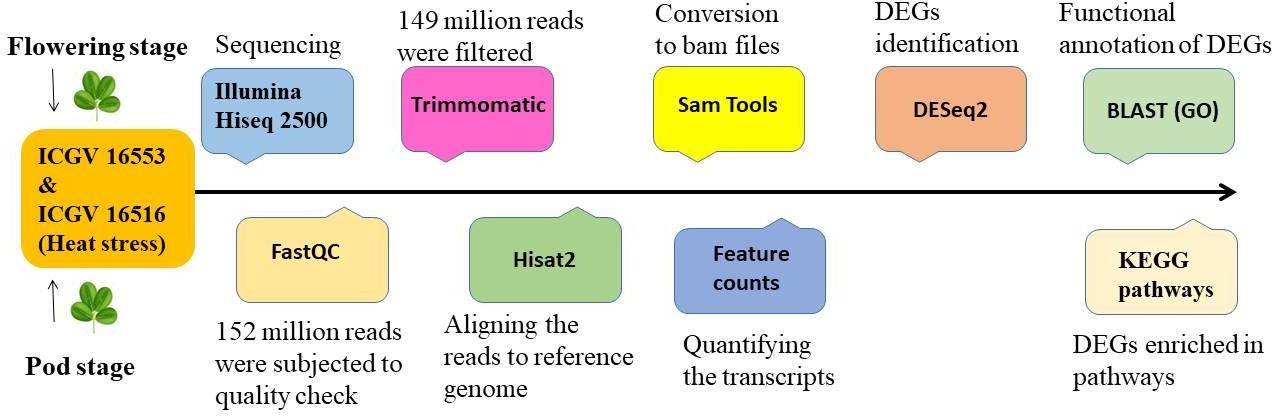


**Supplementary Figure S1: RNA-Seq workflow from sampling to enriched DEGs in pathways Note:** DEGs- Differentially expressed genes; GO- Gene ontology.


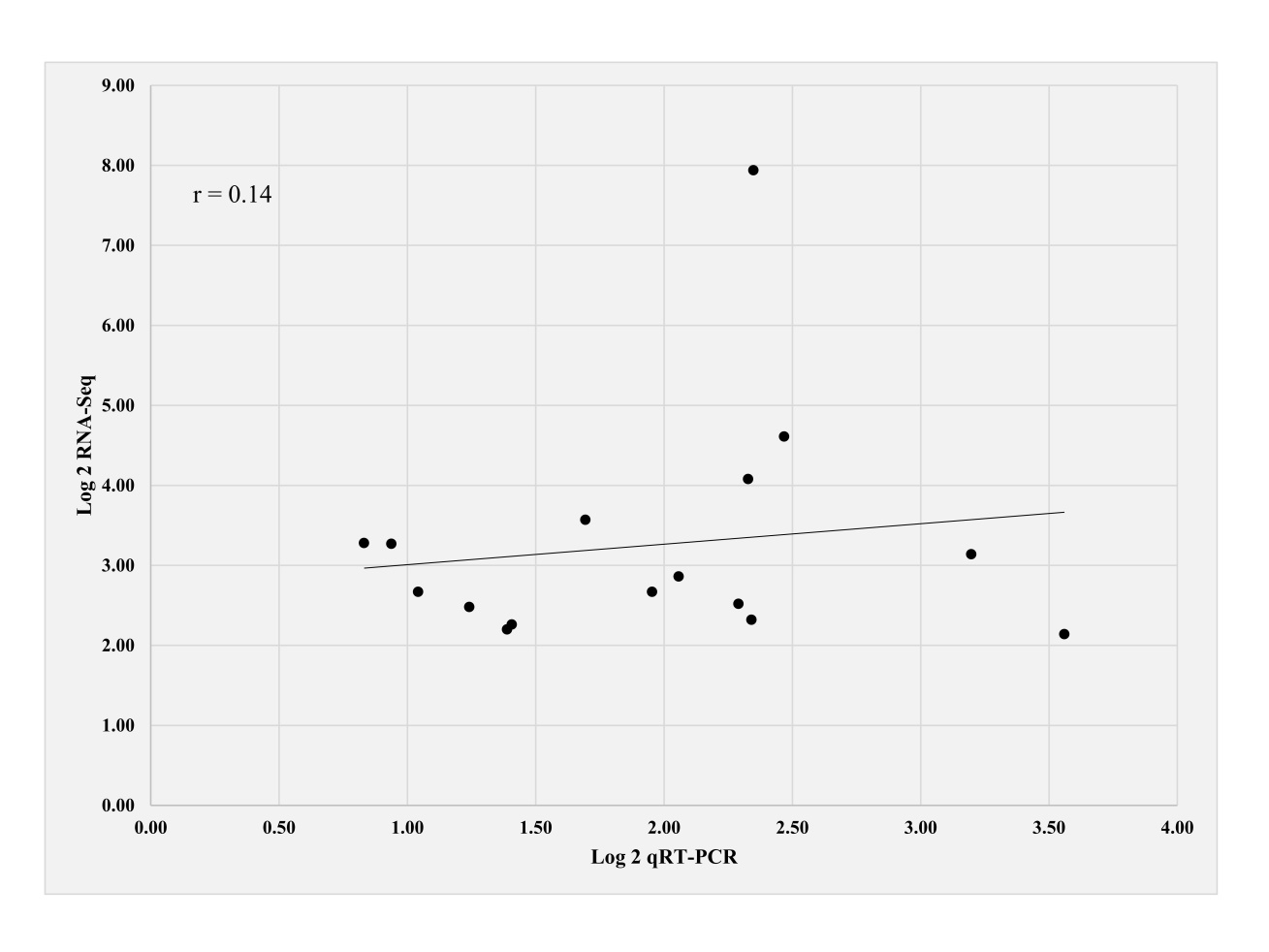


**Supplementary Figure S2: Correlation between selected stress responsive genes analyzed by qRT-PCR and RNA-Seq.**

Note: X-axis represents the fold change values selected stress responsive genes by qRT-PCR; Y-axis represents the fold change values selected stress responsive genes by RNA-Seq.
